# Fitness costs of increased cataract frequency and cumulative radiation dose in natural mammalian populations from Chernobyl

**DOI:** 10.1038/srep19974

**Published:** 2016-01-27

**Authors:** Philipp Lehmann, Zbyszek Boratyński, Tapio Mappes, Timothy A. Mousseau, Anders P. Møller

**Affiliations:** 1 Centre of Excellence in Biological Interactions Research, Department of Biological and Environmental Science, P.O. Box 35, FI-40014 University of Jyväskylä, Finland; 2 Department of Zoology, SE-106 91 University of Stockholm, Sweden; 3 CIBIO/InBIO, Research Center in Biodiversity and Genetic Resources, University of Porto, 4485–661 Vairão, Portugal; 4 Department of Biological Sciences, University of South Carolina, Columbia, SC 29208, USA; 5 Laboratoire d’Ecologie, Systématique et Evolution, CNRS UMR 8079, Université Paris-Sud, Bâtiment 362, F-91405 Orsay Cedex, France

## Abstract

A cataract is a clouding of the lens that reduces light transmission to the retina, and it decreases the visual acuity of the bearer. The prevalence of cataracts in natural populations of mammals, and their potential ecological significance, is poorly known. Cataracts have been reported to arise from high levels of oxidative stress and a major cause of oxidative stress is ionizing radiation. We investigated whether elevated frequencies of cataracts are found in eyes of bank voles *Myodes glareolus* collected from natural populations in areas with varying levels of background radiation in Chernobyl. We found high frequencies of cataracts in voles collected from different areas in Chernobyl. The frequency of cataracts was positively correlated with age, and in females also with the accumulated radiation dose. Furthermore, the number of offspring in female voles was negatively correlated with cataract severity. The results suggest that cataracts primarily develop as a function of ionizing background radiation, most likely as a plastic response to high levels of oxidative stress. It is therefore possible that the elevated levels of background radiation in Chernobyl affect the ecology and fitness of local mammals both directly through, for instance, reduced fertility and indirectly, through increased cataractogenesis.

A cataract is a clouding of the lens that affects vision by increasing opacity and reducing light transmission to the retina^1^. Thus cataracts reduce visual acuity of the bearer, up to the point of total blindness. In humans, cataracts generally develop as a consequence of ageing, as old lens fibres cease to be removed at the same rate as new lens fibres are being added^2^,^3^. There are, however several extrinsic factors which can lead to the formation of presenile cataracts, such as smoking^4^, ultraviolet radiation^5^ or dehydration^6^. A common denominator is oxidative stress, which is related to several of the aforementioned extrinsic factors and to age, as antioxidant levels decrease with age^7^,^8^,^9^. While relatively well studied in humans, the prevalence of cataracts in wild mammals, and their potential ecological significance (i.e. potential fitness costs) remains unknown. In birds, for which visual clues are key components of their life history strategies, cataracts have a significant negative effect on survival and fitness^10^,^11^.

A major cause of oxidative stress in animals is ionizing radiation^12^. Ionizing radiation produces free radicals that consume antioxidant stores and, consequently, lead to oxidative stress^8^. The lenses have been suggested to be particularly vulnerable to the effects of ionizing radiation^12^,^13^, due to the effect of oxidative stress on the high amount of protein that makes up the bulk of the lens^8^. While acute doses of between 0.2 and 0.5 Gy have been shown to lead to detectable changes in human lenses^14^, there is still significant uncertainty of the relationship between acute and chronic radiation dose and cataract formation in humans^13^,^15^ and other mammals. In the present study we investigate if elevated frequencies of cataracts are found in eyes of bank voles (*Myodes* [= *Clethrionomys*] *glareolus*) (Schreiber) collected from natural populations in areas with varying levels of background radiation in Chernobyl Exclusion Zone^16^. In previous studies no major detrimental effects of ionizing radiation have been seen in abundance patterns, life history traits and biopsies of bank voles from Chernobyl^17^,^18^ even though these animals carry significant body burdens of radionuclides^19^ and variation in dorsal fur coloration has been attributed to background radiation^16^. In the present study we tested detrimental effects of ionizing radiation on an important, and susceptible tissue, the lens, and if the possible injuries of lenses are related to the fitness components (e.g. number of offspring) in natural mammalian populations.

## Results

**Figure 1:**
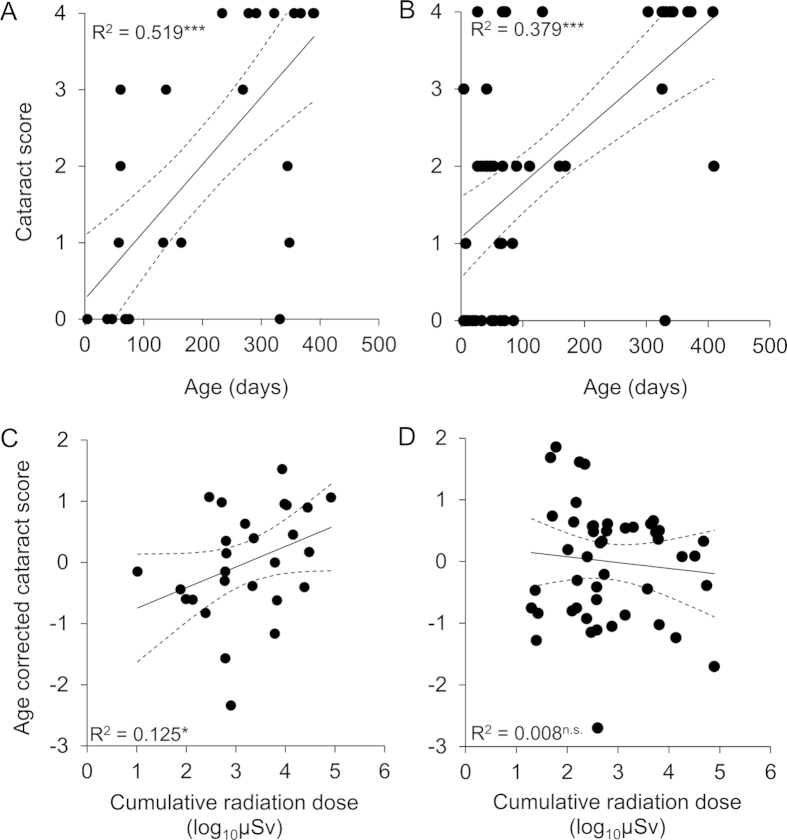
The upper panels show cataract scores regressed against the age estimate for (A) female and (B) male bank voles collected from Chernobyl. The lower panel shows cataract scores corrected for age (standardized residuals from a generalized linear regression on the data split by sex), regressed against the logarithm of lifetime accumulated radiation dose, for (**C**) female and (**D**) male bank voles. The symbol after the R^2^ value denotes the significance level of the regression (n.s. = P > 0.05; *P < 0.05; **P < 0.005; ***P < 0.001). Dashed lines refer to the 95% confidence interval limits of the regression (solid) line.

**Figure 2:**
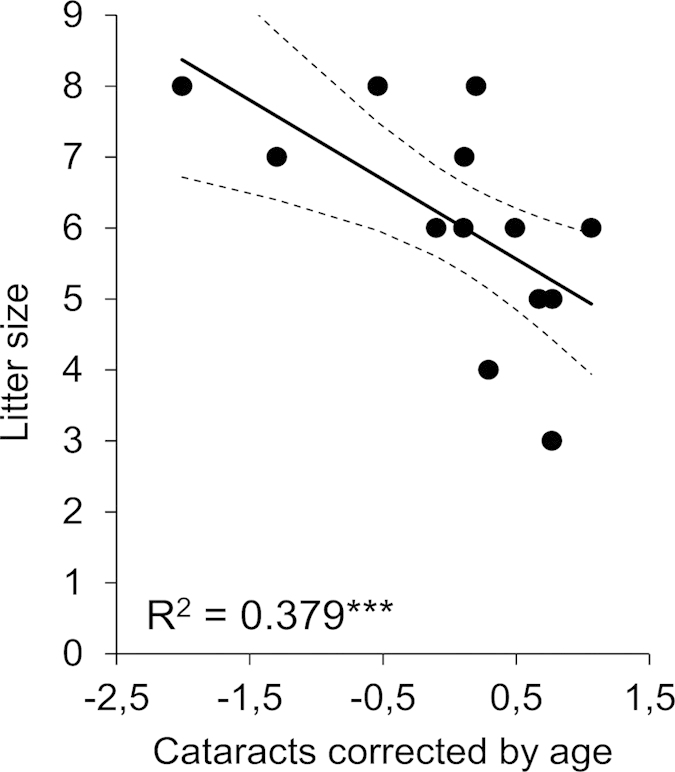
Litter size in female bank voles from Chernobyl was significantly negatively correlated with age and cataract score. The figure shows a regression between litter size and the Pearson residual from a regression between age and cataract score. The symbol after the R^2^ value denotes the significance level of the regression (***P < 0.001). Dashed lines refer to the 95% confidence interval limits of the regression (solid) line.

Cataracts were found in 57 out of 80 voles from Chernobyl (71%). Females and males had cataracts at roughly equal frequency (males: 73%, females 67%, Table 1). While the cataract score increased with age in both sexes, the influence of accumulated radiation dose on cataract score differed between female and male voles (Table 2a). Therefore the data were split by sex and re-analysed. The interaction between age and accumulated radiation dose was not significant in either sex (females: F_1, 23_ = 0.092, P = 0.764; males: F_1, 44_ = 0.396, P = 0.533) and removed from the final model. The frequency of cataracts was positively correlated with age in both females and males (Fig. 1A,B), and age explained a significant amount of the variance in cataract scores in both sexes (Table 2b,c). In addition, the frequency of cataracts was also positively correlated with accumulated radiation dose. However, this effect was only significant for female voles, but not for the male voles (Table 2b,c, Fig. 1C,D). Finally, in the analysis of female litter size the interaction between cataract score and age was non-significant and removed (F_1, 10_ = 4.078, P = 0.071) after which the analysis was redone with only the main effects. Number of offspring in pregnant female voles was positively correlated with age and strongly negatively correlated with cataract score (Full model: F_2, 11_ = 14.916, P = 0.001; Age: F_1, 11_ = 23.194, P = 0.001; Cataract score: F_1, 11_ = 25.901, P < 0.001; Random factors: estimate ± residual: 0.105 ± 0.111, z = 0.941, P = 0.347, Fig. 2). Overall all results stay unchanged if only animals from 2011 (the majority of samples) were analysed (data not shown).

## Discussion

Significant frequencies of cataracts were found in voles collected from different areas in Chernobyl. The frequency of cataracts increased as a function of age, which either could be due to cumulative exposure to background radiation with age, or an inherent effect of ageing, such as, for instance, decreased antioxidant production^7^,^8^,^9^. A complicating factor is that the relationship between age and radiation sensitivity might be non-linear, as has been shown in rats^20^. The results nevertheless suggest that even though no major detrimental effects of ionizing radiation are seen in population (or geographic) genetic structure or life-history traits of bank voles^17^,^18^,^19^, but see^20^, ionizing radiation does have a significant negative effect on bank voles, seen through the induction of severe cataract formation.

While voles of both sexes had significant cataract frequencies the sexes differed in their responses to local radiation. The cataract score in female voles was positively correlated with the accumulated radiation dose, which further supports the notion that cataracts primarily are gained as a function of accumulated ionizing background radiation^7^,^21^. That the correlation with accumulated radiation dose was only significant for females could be due to several reasons, such as higher baseline oxidative stress levels associated with reproductive investment^22^,^23^, but see^24^. Also female voles that were not pregnant at the time of dissection, in general had mating scars indicating previous mating efforts. In humans it has been suggested that females in general have higher cataract prevalence than males^25^,^26^,^27^. It is also possible that male voles suffer more strongly from cataracts, due to higher movement activity and increased predation risk^28^, than females and therefore, due to high mortality, were under-represented in the current dataset. However, since females show a strong negative relationship between cataract score and fecundity (Fig. 2), the first hypothesis, stronger negative effects on females as a function of increased reproductive effort, seems more plausible. It must be stated that increased radiation might also directly lower fertility^29^,^30^ and indeed, also lifetime accumulated radiation was negatively correlated with fecundity (results not shown). Thus there might be multiple effects on fertility due to radiation. Finally it should be noted that while the effect of radiation was significant only in females, lens deformities were discovered in the majority of male voles (73%). The average background radiation level in Europe varies, but is around 0.27 μSv/h^12^. Compared to that, most areas, even relatively clean ones, added significant background radiation to the average (the median value of 0.274 μSv/h amounts to a 50% increase in the level of ionizing background radiation). Thus, even though no correlation was seen in male voles between age corrected cataract score and local background radiation, the overall high frequency of cataracts does support the notion that also male voles in Chernobyl suffer from increased prevalence of lens deformities. It must finally be stated that short-lived animals such as voles with high general mortality, especially in males, makes partitioning out radiation-specific mortality challenging.

The lifetime accumulated chronic doses of ionizing radiation estimated in the present study for voles, which had lens deformities (cataract score ≥1), were on average 0.01 ± 0.003 Sv (min: 0.0002, max: 0.08). In a study on human children exposed to comparably small cumulative doses of radiation from Chernobyl as in the current study (between 0.029 to 0.086 Sv) a small (3.6%) but significantly increased proportion showed lens changes when compared to non-exposed children^31^, see also^15^. In humans the threshold dose currently estimated for radiation cataractogenesis is 0.5 Sv for acute exposures^32^. Thus, the present study suggests that voles might develop cataracts at lower accumulated radiation doses than humans as a consequence of chronic exposure to ionizing radiation (Table 3). This could be due to several reasons. Firstly, the method used to assess eye deformities in the current study, by microscopically examining dissected lenses, could be more sensitive in detecting small eye lesions than in many large scale human studies where eyes are investigated using non-invasive clinically ascertained methods e.g.^27^ or slit-lamp exams e.g.^15^. Furthermore, since radiation induced cataract formation depends on the rate at which damaged lens epithelial cells divide, differentiate and migrate^33^, small mammals with high metabolic rate and short lifespan might have lower response thresholds than humans. Also in other animal models, such as the mouse and rat, significantly lower response thresholds for cataract formation have been documented than for humans^34^. A second, methodological issue relates to how lifetime accumulated radiation dose was calculated. In the current study the background radiation rate (in μSv/h) was multiplied with estimated lifespan in hours. This yields a dose reflecting accumulated background radiation on a very local scale, since background radiation reflects trap location. While not strongly dispersing, voles can move significant distances in search of mates, while foraging or to find better habitats^28^,^35^. Thus the dose measure might not reflect individual variation to the extent needed for accurate calculations on dose-response thresholds. This remains an important future task. Also more careful partitioning of sex-specific effects is important, since male and female voles differ in several behavioural and life-history characters, including dispersal, home range size and predation risk^28^,^35^,^36^ which could influence how they are exposed to, and accumulate radiation as well as how cataracts influence survival.

The ecological significance of the high frequency of cataracts in voles from Chernobyl is challenging to deduce, since vision is less important than other senses in bank voles. Indeed, sensory dependence differences and its consequences for individual fitness might be a potential explanation for why higher frequencies of cataracts are recorded in bank voles than in live birds from Chernobyl, which rely more on vision^11^. However, the negative correlation between litter size and cataract seen in breeding females suggests that cataracts may have a negative impact on fitness in wild bank voles (Fig. 2) although at this stage we cannot rule out the possibility that radiation exposure affects both traits independently. Given the results presented here for a rodent and past related studies on birds, it is likely that elevated frequencies of cataracts have a profound effect on the ecology of animal populations affected by nuclear accidents.

## Methods

### Study animals, study areas, general morphology and fecundity

Study animals were captured with Ugglan multiple-capture live traps (Grahnab, Sweden) baited with sunflower seeds and potato, during the summers of 2011, 2013 and 2014, from 41 locations in the Chernobyl region of Ukraine^16^. In 2011, 64 voles (26 females, 38 males), in 2013, 12 voles (5 females, 7 males) and in 2014, 12 voles (3 females, 9 males) were captured. Animals were killed by cervical dislocation and stored at −20 °C on site. All procedures were performed in accordance with relevant guidelines and regulations. The study was approved by the Finnish Ethical Committee (license numbers: ESLH-2008-04660/Ym-23 and ESLH-2009-09663/Ym-23). Vole length was measured as the ventral distance between the tip of the snout to the anus of the straightened animal (to the nearest 0.1 mm), while weight of the animal was measured after removal of embryos if present (Mettler Toledo XS204, to the nearest 0.1 g). Number of offspring (embryos) was counted from all pregnant females (14 in total).

### Measurements of background radiation

Soil radiation level at the exact trap location was measured with a hand-held dosimeter (Inspector, SE International, Inc, Summertown, TN, USA) calibrated to measure Sieverts (Sv). The average radiation varied between trapping sites from 0.05 to 59.70 μSv/hour. The actual dose in individuals is likely to be strongly correlated to these “residential” measurements although the effects of ingestion of foods of varying activity levels is likely to increase the variance of doses considerably^37^.

### Detection and quantification of cataracts

Cataracts were identified through visual inspection of lenses dissected from eyes of voles under a preparation microscope (Olympus SZ-CTV). Dissections were carefully performed in distilled water in 2013 (animals collected 2011) and 2014 (animals collected 2013 and 2014) after thawing animals on ice. After removal, the lens was placed on millimetre paper on top of a line. The presence and size of cataracts were divided into three categories: 0 = no cataracts, line on paper clearly visible, 1 = small cataract, line partially visible, 2 = large cataract, line not visible^10^. Eye scores were combined, and thus each animal got a cataract score from 0 to 4, with 0 representing no cataracts and 4 representing large cataracts in both eyes. Uncertain cases (13 animals with eye or head damage) were removed from analysis. No bias between the right and left eye was seen (paired sample t-test: t_74_ = −0.363, P = 0.718).

### Estimation of age and lifetime accumulated radiation

For age determinations the first molar of the right and left mandibles was removed and the length of the oral root from the upper part of the neck of the tooth measured under a calibrated stereomicroscope (Olympus SZ61) and converted to mm at a resolution of 0.01 mm. Since teeth wear out with time, this measure is strongly positively correlated with age in bank voles^36^,^38^. We estimated the age of voles in hours with the following equation, from^38^:


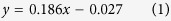


where y is root length in mm and x the age in months (30 day periods). The lifespan was converted from months to hours and multiplied with radiation in μS/h to produce an estimate of lifetime accumulated radiation.

### Statistical analyses

The data was first analysed with a generalized linear mixed model with cataract score as dependent variable, sex as factor and age (in hours) and the logarithm of accumulated radiation dose (log_10_μSv) as covariates. The main effects as well as the interactions age*radiation and sex*radiation were included. Collection year and location were added as random (block) factors. Following this analysis the data were split by sex, and the cataract score then re-analysed as described above. The non-significant interaction between age*accumulated radiation was removed from the final models^39^. A generalized linear mixed model was employed since the cataract score data was slightly overdispersed due to zero-inflation. The influence of cataracts on number of offspring in reproducing females was tested with a generalized linear mixed effect model. Number of offspring was included as target variable, while age and cataract score were added as explanatory variables. Collection year and location were added as random (block) factors. All statistical tests were performed with the IBM SPSS 20.0 software (IBM SPSS Inc., Chicago, IL, USA).

## Additional Information

**How to cite this article**: Lehmann, P. *et al.* Fitness costs of increased cataract frequency and cumulative radiation dose in natural mammalian populations from Chernobyl. *Sci. Rep.* **6**, 19974; doi: 10.1038/srep19974 (2016).

## Figures and Tables

**Table 1:** Descriptive data on morphological traits, cataract score and average background radiation levels in the collection areas and lifetime accumulated radiation dose.

	Total *N*	Length (mm)	Weight (g)	Molar length (mm)	Cataracts (score*)	Radiation (μSv/hour)	Accumulated radiation (μSv)
Females	34	92.9 ± 1.2	22.5 ± 0.8	1.2 ± 0.2	2.0 ± 0.3	2.5 ± 0.8	8020 ± 2924
Males	54	90.8 ± 1.1	22.2 ± 0.6	0.9 ± 0.1	2.1 ± 0.2	1.4 ± 0.3	6909 ± 2317

Years and locations were pooled. Numbers are expressed as mean ± standard error.

*0 = no cataract in either eye, 4 = large cataract in each eye.

**Table 2:** Generalized linear mixed models testing the effect of age, sex and level of accumulated radiation dose (ARD) on the presence and extent of cataracts in lenses of bank voles from Chernobyl.

Effect	df_(n,d)_	*F*	*P*
a) *Full model*			
Corrected model	**5, 69**	**14.947**	**<0.001**
Sex	**1, 69**	**7.437**	**0.008**
Age	**1, 69**	**7.387**	**0.008**
ARD	1, 69	1.323	0.254
Sex* ARD	**1, 69**	**5.371**	**0.023**
Age*ARD	1, 69	0.270	0.605
b) *Female*			
Corrected model	**2, 24**	**20.094**	**<0.001**
Age	**1, 24**	**18.761**	**<0.001**
ARD	**1, 24**	**5.545**	**0.027**
c) *Male*			
Corrected model	**2, 45**	**15.566**	**<0.001**
Age	**1, 45**	**26.723**	**<0.001**
ARD	1, 45	0.600	0.443

(a) includes all data, while (b) and (c) show results for the data split by sex.

Three parameters and their interactions were estimated using restricted maximum-likelihood procedures. Sampling year and location were included as random factors in each of the models. For a) estimate ± residual: 1.313 ± 0.253, z = 5.187, *P* < 0.001, b) 1.240 ± 0.385, z = 3.221, *P* = 0.001 c), 1.540 ± 0.331, z = 4.654, *P* < 0.001. df_(n,d)_, degrees of freedom from numerator (n) and denominator (d); F, test statistic, P, probability, significant values (P < 0.05) are shown in bold.

**Table 3:** Summary of results from studies measuring eye deformities in response to low doses of radiation compared to current study.

Reference	Species	Time since exposure	Numbers in cohort	Dose range (Sv)	Odds ratio Sv^−1^ (lower, upper 95% CI)
Hsieh et al. 2010^15^	*Homo sapiens*	7 years	73	0.005–0.06	Increased risk, but OR not calculated.
Day et al. 1995^31^	*Homo sapiens*	5–7 years	991	0.030	Increased risk, but OR not calculated.
Chodick et al. 2008^40^	*Homo sapiens*	~19 years	35705	0.005–0.06	1.98 (-0.69, 4.6)^a^
Neriishi et al. 2007^41^	*Homo sapiens*	55–57 years	3761	0–3	1.39 (1.24, 1.55)
Worgul et al. 2007^42^	*Homo sapiens*	12–14 years	8607	0–1	1.65 (1.18, 2.30)
Rafnsson et al. 2005^43^	*Homo sapiens*	Working life	445	0–0.48	3.02 (1.44, 6.35)
Markiewicz et al. 2015^44^	*Mus musculus*	1-10 months	144	0–2	Dose response peak at 0.5 Sv^b^, but OR not calculated.
Current study	*Myodes glareolus*	Chronic exposure	88	0.0002–0.08	1.69 (0.81, 3.53)^c^

^a^Excess relative risk.

^b^At 500 mGy.

^c^The logarithm (log_10_) of the predictor, life-time accumulated background radiation in μSv, was used to generate the odds ratio.
